# Contributions to the History of Development of the Teeth

**Published:** 1888-04

**Authors:** Carl Heitzmann, C. F. W. Bödecker


### Independent Practitioner.

VOL. IX. APRIL, 1888. NO. 4.

NOTE.—No paper published or to be published in another journal will be accepted for this department. All papers must be in the hands of the Editor before the first day of the month preceding that in which they are expected to appear. Extra copies will be furnished to each contributor of an accepted original article, and reprints, in pamphlet form, may be had at the cost of the paper, press-work and binding, if ordered when the manuscript is forwarded. The Editor and Publishers are not responsible for the opinions expressed by contributors. The journal is issued promptly, on the first day of each month.

### Original Communications.

### CONTRIBUTIONS TO THE HISTORY OF DEVELOPMENT OF THE TEETH.

BY CARL HEITZMANN, M. D., AND C. F. W. BÖDECKER, D. D. S., M. D. S.

CONTINUED FROM PAGE 121.

Horace M. Hayden (American Journal of Dental Science, First Series, Vol. I, page 137,) (From the Medical Repository for 1813) was of opinion that a membrane, which he calls the semi-osseous stratum, analogous to that which lines the shell of an egg, was present between the enamel and the dentine, for the purpose of holding the two substances together. This author, at great length, quotes and criticises the assertions of Blake, which were first published in 1798 and 1801. Hayden questions the possibility of the formation of the enamel by the action of a membrane, especially as it (the enamel organ) does not contain vessels which furnish it with red blood, and he, therefore, rather favors the theory of Hunter. He is of opinion the enamel is formed by the semi-osseous stratum, that the more subtile parts of the ossific matter of the tooth are infiltrated, strained off, or deposited, which, when crystallized, forms the enamel or cortex striatus. This author also denies that the cementum is formed by a membrane, and believes that there is proof positive of its being deposited only by the arteries that are distributed upon the membrane. He then speakes of caries of the dentine, and the reaction which, during this process, occurs in the pulp-chamber of a living tooth, which he also attributes to the action of the blood-vessels, and not to any kind of membrane. He entertains like views regarding the formation of the dentine.

Th. Schwann (Microscopische Untersuchungen, Berlin, 1839, page 117), after extracting the lime salts from the enamel of a fœtal tooth, obtained an organic residue which, under the microscope, appeared like the forms of enamel prisms, and thus came to the conclusion that the enamel must be either an ossification of this organic substance, or the organic substance must be present in the pores of the enamel prisms. He gives a good description of the enamel cells, and observed that they arise from the round cells, as he noticed near the summit of the crown in a preparation these cells to be cylindrical, whereas, toward the neck they were round. He also mentions the occurrence of a peculiar substance present between the enamel membrane and the formed enamel. In regard to the formation of the enamel, he gives these theories, the last of which he regards as the most probable, and this is, that the prismatic cells separate from the enamel membrane, grow into the enamel, and then become ossified, which, as he states, brings this theory in harmony with the development of other tissues.

The dentine, which he calls the peculiar substance of the tooth, is made up of canaliculi and a structureless basis-substance. The canaliculi he believed to be empty, from the fact that he succeeded in coloring them with ink. In reference to the formation of the dentine, Schwann states that he is unable to give a definite account of it, and after quoting Purkinje and Raschkow, admits that he cannot agree with them, and further on states that we have to regard the dentine as composed of fibers (basis-substance), between which the canals, possessed of separate walls, are present.

But he asks, in what relations stand the fibers and the canaliculi to the cells (odontoblasts), and believes it possible that the dentine is the ossified substance of the pulp. He also states that as the cylindrical cells (odontoblasts) are ossified, they are replaced by others, which are formed out of the round cells of the pulp. Schwann then returns to the question, what are the dentine canaliculi, and states that at first he regarded them as indentical with the canaliculi in bone-tissue, which are, as Schwann says, continuations of the bone-cells. This author noticed fine fibers upon the surface of the pulp of pig's teeth, which were attached to the cylindrical cells, and which, in their position, corresponded to the dentinal canaliculi. But he was unable to observe such formations in human teeth, and therefore abandoned this theory.

In regard to the bony substance (cementum), Schwann states that it is indentical with the ordinary bone tissue, and thus requires no special explanation.

Kollmann (Sitzungsberichte der K. B. Academie der Wissenschaften zu München, 1869, page 162) asserts that the enamel cuticle is present on every tooth, and even upon those of adult persons which have been in use for many years. The enamel cuticle, (Schmelzoberhäutchen,) according to the views of Kollmann, is the youngest layer of enamel. (This the authors have depicted on page 284, Vol. VIII, Fig. 8.) This cuticle in the embryonal state is situated between the newly calcified enamel and the ameloblasts, while upon the crowns of the teeth which have penetrated the gums it is represented by the outermost layer of enamel which is covered by another (Nasmyth's) membrane, and which, according to Kollmann and Erdl,* is nothing but a thin layer of flat epithelia derived from the mucous membrane of the gum. (The same view has been entertained by the writers.) Kollmann further states that each enamel cell is enveloped by a thin membrane, and this membrane is very distinct at the free margins, where it is from $\frac{1}{1000}$ to $\frac{1}{1200}$ m.m. thick, and covers the newly formed enamel in the form of a light streak, which, after the whole enamel is formed, is itself calcified and remains upon the enamel as "Schmelzoberhäutchen." Kollmann regards this membrane, which, by Huxley, was called "membrana præformativa," to be the specific organ which excretes the

* Münchner Academie Abhandlungen Math. Nat. Klasse, 1841, page 515.

enamel, and states distinctly that the formation of the enamel grows on under this membrana præformativa (Schmelzoberhäutchen).

Emil Dursy (Zur Entwicklungsgeschichte des Kopfes des Menschen und der höheren Wirbelthiere, Tubingen, 1869, page 211) gives a good description of the formation of the primitive fold, and also mentions the occurrence of epithelial nests derived from the external epithelium.

Thomas H. Huxley (Quarterly Journal of Microscopical Science, London, 1853, page 149) confines himself to the description of the pulp, the capsule and the enamel organ, and their relations to the dentine, enamel and cementum, as well as the soft tissues contained in them. In regard to the pulp, he opposes the idea of Goodsir, but is of opinion that it grows upward from the basement membrane of the oral mucosa, and is in connection with the tooth capsule. The epithelium (enamel-organ) is situated between these two formations, as stated by Huxley. In reference to the relations of the enamel, dentine and cementum to the above formation, this author states: "Neither the capsule nor the 'enamel-organ' takes any direct share in the development of the dental tissues, all three of them—viz., enamel, dentine and cement—being formed beneath the membrana præformativa, or basement membrane of the pulp." These assertions were based upon the fact that when strong acetic acid was applied to a specimen from a human fœtus of seven months of age, a membrane about $\frac{1}{1500}$ to $\frac{1}{1600}$ of an inch in thickness became detached. He further states "that at its lower edge this membrane gradually loses all structure, and passes into the membrana præformativa. In fact, it is the altered membrana præformativa itself." In regard to the development of the dentine this author states "that the pulp is not converted directly into the dentine, and that the structure of the latter does not depend upon the calcification of pre-existing elements." On the development of the enamel, he further states: "Still less can the enamel be produced by any conversion of a cellular structure. Between it and anything which can be called a nucleated cell, it has on the outer side Nasmyth's membrane; on the inner, the layer of dentine, which in man is formed before it. The fibers of which it is composed are structureless, and almost horny; and I think we must be content for the present to consider its existence and its structure as ultimate facts, not explicable by the cell theory." On the cementum we find that Huxley has noticed the striated appearance of the neck of this tissue as mentioned in the *Dental Cosmos*, 1879, page 650, as he states: "In a morphological point of view, the relations of the cement show it to be homologous with the enamel." * * * The upper portion of the cement exhibits in places a very distinct transverse striation, resembling its perfect enamel." This author also mentions that Nasmyth's membrane is identical with the structure of the enamel, but at the same time states that it is certain that this layer is cementum.

His researches are concluded as follows :

1. "The teeth are true dermal structures, formed by the deposition of calcareous matter beneath the basement membrane of a dermal papilla, or that which corresponds with one."

2. "Neither the capsule nor the enamel organ, which consists of the epithelium of both the papilla and the capsule, contribute directly in any way to the development of the dental tissues, though they may indirectly."

3. "The histological elements of the pulp take no direct part (except, perhaps, eventually in the cement) in the development of the dental tissues, becoming either absorbed or being pressed in by the gradual increase of the latter. The Conversion Theory is, therefore, as incorrect as the Excretion Theory, and the dentine is formed, not by ossification of the histological elements of the pulp, but by deposition in it," *parenchymate materiam suppeditante.* After which the author refers to the analogy of the teeth to the hair.

George Rolleston (Quarterly Journal of Microsc. Science, London, 1872, page 109), on the development of the teeth of mammals, observed blood vessels to be present in the stellate reticulum of the enamel organ at the time when the formation of the enamel is most active. He regards the stellate tissue to be the matrix of the enamel cells, but does not mention anything more about the formation of the enamel.

E. Mühlreiter (Deutsche Vierteljahrsschr. für Zahnheilkunde, 1868, page 168) only examined human teeth after shedding, and is of opinion that the formation of dentine is accomplished in the same manner in a young growing tooth as in its earlier stage when still within the tooth-sac. This author believes the odontoblasts to be composed of two distinct layers of cells. One set is in direct connection with the dentinal fibers; the other is directed with their long offshoots toward the central portion of the pulp, and thus the pear- or club-shaped ends of each layer of cells are in loose contact. He derived at this conclusion from the fact that when a freshly extracted tooth was split, and the pulp removed, he never could find a single process upon the surface of the pulp, while the surface of the pulp-chamber exhibited the majority of odontoblast cells, in situation, with their processes extending into the dentinal canaliculi. Regarding the formation of the basis-substance of the dentine, he indorses the opinions of Kölliker and Lent, viz., that it is a secretion derived from the odontoblasts, which gradually elongate and become the dentinal fibers, but he states that in this respect he goes even further than Kölliker, believing that the excreting process is really accomplished by the vessels of the pulp, and that the odontoblasts are only passively engaged in the formation of the basis-substance of the dentine.

Ch. Legros and E. Magitot (The Origin and Formation of the Dental Follicle. Translated by M. S. Dean, Chicago, 1880) say as to the origin of the "stellate cells" of the enamel organ (page 78): "These starred bodies are formed directly at the expense of the polygonal elements, composing the internal mass of the enamel organ. The process is as follows: The basis-substance interposes itself little by little between these originally small polyhedral cells, and thus their walls lose their mutual contact, except at certain points where they still cohere." (Page 80): * * * "These elements of the enamel organ, notwithstanding their stellate form, must be regarded, therefore, as absolutely epithelial in their nature." Nothing is stated in this book about the development of the enamel, dentine or cementum. (Page 66): "The epithelial proliferations (buds of the external epithelium), according to Kollman and Magitot, may become the enamel germs from which the supernumerary teeth originate."

Ch. Robin and E. Magitot (Journal de Physiologie de Brown-Séquard, 1860-1861) give a theory of follicular evolution, according to which the dental bulb is the first part of the follicle that appears in the depth of the jaws, at the bottom of the grooves. * * * Afterwards the enamel organ is seen; and the wall emanating from the bulb, and rising upon the sides of these organs so as to surround them, and to unite at the apex of the follicle. Magitot, however, in the book above quoted, admitted that the order of genesis was misconceived, and the enamel organ makes its appearance before the bulb (papilla).

(TO BE CONTINUED.)

